# Cyclin D1 overexpression is an indicator of poor prognosis in resectable non-small cell lung cancer

**DOI:** 10.1038/sj.bjc.6690661

**Published:** 1999-09

**Authors:** J S Keum, G Kong, S C Yang, D H Shin, S S Park, J H Lee, J D Lee

**Affiliations:** Department of Pathology, School of Medicine, Sungkyunkwan University, # 108, Pyung-Dong, Seoul, 110-102, Korea; Departments of Pathology and Internal Medicine, College of Medicine, Hanyang University, # 17, Haengdang-Dong, Seoul, 133-791, Korea

**Keywords:** non-small-cell lung cancer, cyclin D1, immunohistochemistry, progression, prognosis

## Abstract

Cyclin D1 is one of the G1 cyclins that control cell cycle progression by allowing G1 to S transition. Overexpression of cyclin D1 has been postulated to play an important role in the development of human cancers. We have investigated the correlation between cyclin D1 overexpression and known clinicopathological factors and also its prognostic implication on resected non-small-cell lung cancer (NSCLC) patients. Formalin-fixed and paraffin-embedded tumour tissues resected from 69 NSCLC patients between stages I and IIIa were immunohistochemically examined to detect altered cyclin D1 expression. Twenty-four cases (34.8%) revealed positive immunoreactivity for cyclin D1. Cyclin D1 overexpression is significantly higher in patients with lymph node metastasis (50.0% vs 14.4%, *P* = 0.002) and with advanced pathological stages (I, 10%; II, 53.8%; IIIa, 41.7%, *P* = 0.048; stage I vs II, IIIa, *P* = 0.006). Twenty-four patients with cyclin D1-positive immunoreactivity revealed a significantly shorter overall survival than the patients with negativity (24.0 ± 3.9 months vs 50.1 ± 6.4 months, *P* = 0.0299). Among 33 patients between stages I and II, nine patients with cyclin D1-positive immunoreactivity had a much shorter overall survival (29.7 ± 6.1 months vs 74.6 ± 8.6 months, *P* = 0.0066). These results suggest that cyclin D1 overexpression is involved in tumorigenesis of NSCLCs from early stage and could be a predictive molecular marker for poor prognosis in resectable NSCLC patients, which may help us to choose proper therapeutic modalities after resection of the tumor. © 1999 Cancer Research Campaign


British Journal of Cancer (1999) **81**(1), 127–132
© 1999 Cancer Research Campaign
Article no. bjoc.1999.0661

### Cyclin D1 overexpression is an indicator of poor prognosis in resectable non-small cell lung cancer

**JS Keum[1], G Kong[2], SC Yang[3], DH Shin[3], SS Park[3], JH Lee[3] and JD Lee[2]**

[1]Department of Pathology, School of Medicine, Sungkyunkwan University, # 108, Pyung-Dong, Chongro-Ku, Seoul, 110-102, Korea; Departments of [2]Pathology and [3]Internal Medicine, College of Medicine, Hanyang University, # 17, Haengdang-Dong, Sungdong-Ku, Seoul, 133-791, Korea

**Summary** Cyclin D1 is one of the G1 cyclins that control cell cycle progression by allowing G1 to S transition. Overexpression of cyclin D1 has been postulated to play an important role in the development of human cancers. We have investigated the correlation between cyclin D1 overexpression and known clinicopathological factors and also its prognostic implication on resected non-small-cell lung cancer (NSCLC) patients. Formalin-fixed and paraffin-embedded tumour tissues resected from 69 NSCLC patients between stages I and IIIa were immunohistochemically examined to detect altered cyclin D1 expression. Twenty-four cases (34.8%) revealed positive immunoreactivity for cyclin D1. Cyclin D1 overexpression is significantly higher in patients with lymph node metastasis (50.0% vs 14.4%, $P = 0.002$) and with advanced pathological stages (I, 10%; II, 53.8%; IIIa, 41.7%, $P = 0.048$; stage I vs II, IIIa, $P = 0.006$). Twenty-four patients with cyclin D1-positive immunoreactivity revealed a significantly shorter overall survival than the patients with negativity (24.0 ± 3.9 months vs 50.1 ± 6.4 months, $P = 0.0299$). Among 33 patients between stages I and II, nine patients with cyclin D1-positive immunoreactivity had a much shorter overall survival (29.7 ± 6.1 months vs 74.6 ± 8.6 months, $P = 0.0066$). These results suggest that cyclin D1 overexpression is involved in tumorigenesis of NSCLCs from early stage and could be a predictive molecular marker for poor prognosis in resectable NSCLC patients, which may help us to choose proper therapeutic modalities after resection of the tumor.

**Keywords** non-small-cell lung cancer; cyclin D1; immunohistochemistry; progression; prognosis

The incidence of lung cancer has been increasing while death rates from cancer have declined in most developed countries during the past 20 years. In the USA it is the leading cause of death as a result of a more than threefold increase during the same time period. Although there has been a recent decline in the USA and Canada, the death rate from lung cancer in developing countries continues to accelerate (de Vita et al, 1997).

Because relapse is frequent after resection of even early-stage non-small-cell lung cancer (NSCLC), the long-term survival rate remains disappointingly poor (de Vita et al, 1997). The two major prognostic factors for patients with early-stage resectable cancers are the size of the tumour and the presence or absence of lymph node metastasis (Mountain, 1986; Naruke et al, 1988). Histological subtype may provide some additional prognostic information; outcomes of large-cell lung cancers are the least favourable subtype while resected squamous cell carcinomas and adenocarcinomas have a more favourable prognosis (Kayser et al, 1987; Mountain et al, 1987). Other poor prognostic factors include lack of tumour differentiation, lymphatic vessel invasion in patients with metastasis-free lymph nodes, blood vessel invasion, high mitotic index, loss or alteration of the expression of blood group antigens on the tumour cells, the presence of tumour-associated carbohydrate antigens, aneuploid y, high S phase fraction on flow cytometry, increased proliferating cell nuclear antigen (PCNA) index, elevated level of serine proteases like urokinase and plasminogen, and a high microvessel count (Rosvold, 1996).

With the advance in molecular biology some genetic changes have been suggested to be useful prognostic markers. Mutations in K-*ras* (Mitsudomi et al, 1991), overexpression of c-*erb*B-2 protein (Kern et al, 1990, 1994; Tateishi et al, 1991), mutation of p53 (Mitsudomi et al, 1993) and lack of Rb1 (Xu et al, 1994) or bcl-2 protein expression (Pezzella et al, 1993; Fontanini et al, 1995) have all been reported as adverse prognostic factors.

Recently, in addition to mitogens and tumour suppressor genes, some of the essential components of cell cycle machinery itself have been found to show oncogenic potential (Motokura and Arnold, 1993; Lovec et al, 1994). Among them, cyclin D1 appears to be the most strongly implicated in tumorigenesis. Cyclin D1 has a regulatory role in the G1 to S transition of the cell division cycle. There have been genetic evidences of tumour-specific rearrangements and amplifications of the cyclin D1 gene in the experimental-induced malignant phenotype (Bartkova et al, 1994; Lovec et al, 1994; Lukas et al, 1994; Wang et al, 1994).

The role of cyclin D1 protein in human cancers has recently been able to be performed in a large scale using a monoclonal antibody specific for this oncoprotein with an optimizing protocol for immunohistochemical detection of cyclin D1 on archival tissue sections (Gillett et al, 1994; Bartkova et al, 1995). Some of the human primary cancers have been found to have amplification or overexpression of the relevant gene locus of cyclin D1 including breast (Gillett et al, 1994; McIntosh et al, 1995; Simpson et al, 1997), oesophagus (Jiang et al, 1992; Adelaide et al, 1995), liver

*Received 14 August 1998*
*Revised 27 January 1999*
*Accepted 11 March 1999*

*Correspondence to:* G Kong

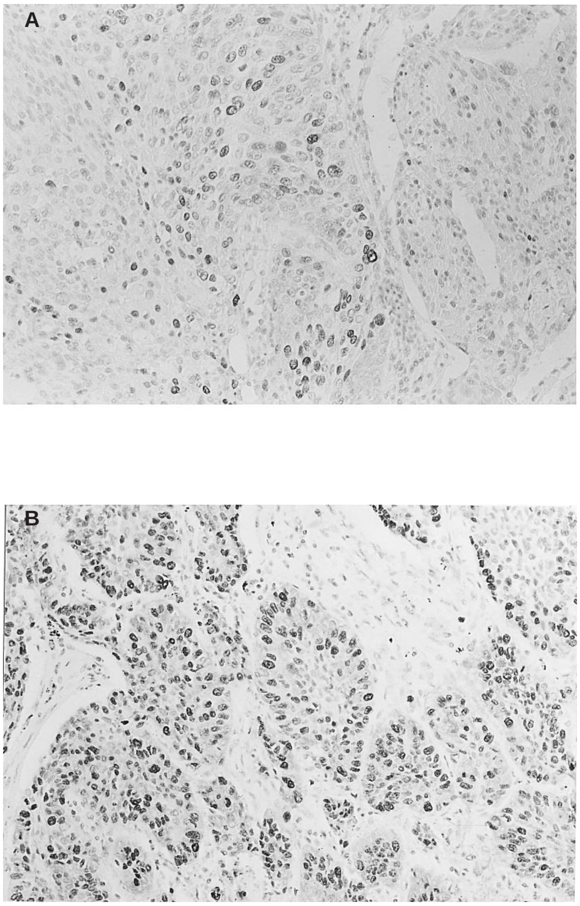

**Figure 1** Immunohistochemical stainings of poorly differentiated squamous cell carcinoma for cyclin D1 (**A**) and PCNA (**B**) show distinct intranuclear immunoreactivity

(Zhang et al, 1993; Nishida et al, 1994), head and neck (Callender et al, 1994, Michalides et al, 1995) and urinary bladder cancers (Proctor et al, 1991; Shin et al, 1997).

A few studies have reported that overexpression of cyclin D1 is implicated in the tumorigenesis of NSCLCs (Betticher et al, 1996; Kwa et al, 1996; Mate et al, 1996; Caputi et al, 1997). However, there has been controvery and limited available data for the significance of cyclin D1 overexpression on the prognosis of NSCLC patients. In the present study, we have investigated whether immunohistochemically detected cyclin D1 overexpression is associated with the survival of patients with early-stage resectable NSCLCs.

### MATERIALS AND METHODS

### Tumour samples

The tumour specimens were obtained from lobectomies or pneumonectomies performed at the Hanyang University Hospital placed in Seoul, Korea between 1985 and 1994. Consecutive 69 NSCLC patients with resectability were selected after pathological examination of the formalin-fixed and paraffin-embedded tumour specimens (stage I–IIIa) (stage I (T1–2 N0 M0), stage II (T1–2 N1 M0), stage IIIa (T1–3 N2 M0, T3 N0 M0, T3 N1 M0)) (Mountain, 1986). The patients had been followed up through a period ranging from 0.5 to 108 months after resection of the primary tumour.

### Immunohistochemical study

Immunohistochemical staining was performed by the method previously described (Shin et al, 1997). Briefly, representative tissue sections from 69 cases were deparaffinized and rehydrated. After boiling the slides in a microwave oven (containing 0.01 M sodium citrate buffer pH 6.0; 800 W), endogenous peroxidase was blocked with 3% hydrogen peroxide–methanol. After washing with cold 0.5 M Tris-buffered saline (TBS), non-specific binding was inhibited by incubation with normal goat serum (Dako, Carpenteria, CA, USA) for 20 min. Monoclonal mouse antihuman cyclin D1 (P2D11F11, 1:200 in dilution, Novocastra, Newcastle, UK) and PCNA antibodies (PC10, 1:100 in dilution; Dako) were applied and incubated for 30 min. After washing with TBS three times, the sections were incubated with biotinylated antimouse IgG (Dako) for 30 min. After washing, peroxidase–antiperoxidase conjugate (Dako) was applied. They were then stained with diaminobenzidine tetrahydrochloride (Dako) and counterstained with Meyer's haematoxylin. Positive control was identified on the sections from formalin-fixed and paraffin-embedded WI-38 cells (ATCC, Rockville, MD, USA) for cyclin D1. Negative control was accompanied by applying phosphate-buffered saline instead of cyclin D1 and PCNA antibodies.

### Analysis

Immunohistochemical reactivity of cyclin D1 was interpreted as positive when there was distinct nuclear staining with a brownish tincture in more than 5% of the tumour cell population (Michalides et al, 1995; Shin et al, 1997) (Figure 1A). For PCNA, distinct nuclear staining with a brownish tincture was considered as positive and the index of PCNA was calculated in percentage of positive cells among approximately 1000 counted tumour cells (Figure 1B).

The correlation of cyclin D1 immunoreactivity with clinical factors such as age, sex, histopathological type, stage of the tumour, extent of the primary tumour, metastatic status of the lymph node and PCNA index was analysed through the Mann–Whitney *U*-test or analysis of variance (ANOVA) according to the characteristics of the data. Overall survival in relation to cyclin D1 immunoreactivity was analysed with the Kaplan–Meier method, and their difference with the log-rank test. Multivariate analysis for survival was assessed using a forward step-wise Cox regression model. The variables included tumour (T) and nodal status of TMN system, stage of the tumour and cyclin D1 positivity. The statistical analysis was performed using the SPSS 7.5 software package (SPSS Inc., Chicago, IL, USA). $P < 0.05$ was considered statistically significant.

### RESULTS

### Clinical features

Among 69 patients, 53 were men and 16 were women. The age difference ranged from 40 to 73 years (mean 57.2 years). The clinical follow-up period was between 0.5 months and 108 months. Twenty cases were in stage I, 13 in stage II and 36 in stage IIIa. There were 41 squamous cell carcinomas, 21 adenocarcinomas and seven large-cell undifferentiated carcinomas. Two giant cell carcinomas were included in the latter group. Five were T1, 43 T2 and 21 T3 in accordance with the extent of the primary

© 1999 Cancer Research Campaign

**Table 1** Cyclin D1 immunoreactivity and clinicopathological features

| | | Cyclin D1 – | Cyclin D1 + | Positive (%) | *P*-value[a] |
|---|---|---|---|---|---|
| Number of patients | 69 (total) | 45 | 24 | 34.8 | |
| Sex | Male | 38 | 17 | | |
| | | | | | 0.394 |
| | Female | 7 | 7 | | |
| Age | 57.2 ± 8.4 (total) | 57.9 ± 7.4 | 55.7 ± 10.1 | | 0.347[b] |
| Subtype | Squamous | 28 | 13 | 31.7 | |
| | Adeno | 11 | 10 | 47.6 | 0.784 |
| | Large cell | 6 | 1 | 14.3 | |
| Primary tumour tumour | T1 | 5 | 0 | 0 | |
| T2 | 26 | 17 | 39.5 | 0.637 | |
| | T3 | 14 | 7 | 33.3 | |
| Lymph node | N0 | 24 | 3 | 11.1 | |
| metastasis | N1 | 11 | 10 | 47.6 | 0.002 |
| | N2 | 10 | 11 | 52.4 | |
| Stage | I | 18 | 2 | 10.0 | |
| | II | 6 | 7 | 53.8 | 0.048 |
| | IIIa | 21 | 15 | 41.7 | |

[a]Mann–Whitney *U*-test; [b]*t*-test.

**Table 2** Survival functions (Kaplan–Meier) of the 69 patients according to various factors

| | Factor | Number of patients | Survival time[a] (Mean ± S.D.) | Per cent censored | *P*-value[b] |
|---|---|---|---|---|---|
| Sex | Female | 16 | 47.4 ± 10.4 | 31.25 | 0.6129 |
| | Male | 53 | 41.8 ± 5.7 | 30.19 | |
| Tumour type | | | | | |
| | Squamous | 41 | 48.3 ± 6.4 | 41.5 | |
| | Adeno | 21 | 36.2 ± 8.2 | 19.1 | 0.1887 |
| | Large cell | 7 | 27.4 ± 5.5 | 0 | |
| T status | T1 | 5 | 66.9 ± 19.8 | 60.0 | |
| | T2 | 43 | 47.6 ± 6.5 | 34.9 | 0.0133 |
| | T3 | 21 | 24.5 ± 5.6 | 14.3 | |
| N status | N0 | 27 | 66.1 ± 8.1 | 51.9 | |
| | N1 | 21 | 35.8 ± 8.4 | 28.6 | 0.0002 |
| | N2 | 21 | 20.8 ± 3.7 | 4.8 | |
| Stage | I | 20 | 79.18.5 | 65.0 | |
| | II | 13 | 35.78.9 | 30.8 | < 0.00001 |
| | III | 36 | 22.93.7 | 11.1 | |
| Cyclin D1 | – | 45 | 50.1 ± 6.4 | 35.6 | 0.0299 |
| | + | 24 | 24.0 ± 3.9 | 20.8 | |
| Cyclin D1[c] | – | 24 | 74.6 ± 8.6 | 62.5 | 0.0066 |
| | + | 9 | 29.7 ± 6.1 | 22.2 | |

S.D., standard deviation; [a]months; [b]log-rank test; [c]only stage I and II.

tumour. Twenty-seven patients were free of tumour metastasis in both dissected and biopsied lymph nodes. Twenty-one patients showed ipsilateral bronchopulmonary or hilar nodal metastasis and the other 21 patients had metastasis in the ipsilateral subcarinal mediastinal lymph node.

### Immunohistochemical analysis of cyclin D1

Twenty-four tumours (34.8%) revealed positive immunoreactivity for cyclin D1. The relationship between cyclin D1 immunoreactivity and clinicopathological features are summarized in Table 1. Cyclin D1 overexpression was higher in female patients (50%) than in male patients (30.9%), but there was no significant difference between the two groups ($P$ = 0.394). The cyclin D1 immunopositive group was slightly younger than the negative group with no significant difference ($P$ = 0.347). Among the three histopathological subgroups, adenocarcinoma showed the most frequent positivity (47.6%) followed by squamous cell carcinoma and large-cell carcinoma (31.7% and 14.3% respectively). However, no significant difference was present among the three groups ($P$ = 0.784). Although all five T1 tumours showed no immunoreactivity, T2 and T3 tumours revealed 39.5 and 33.3% of

*© 1999 Cancer Research Campaign*

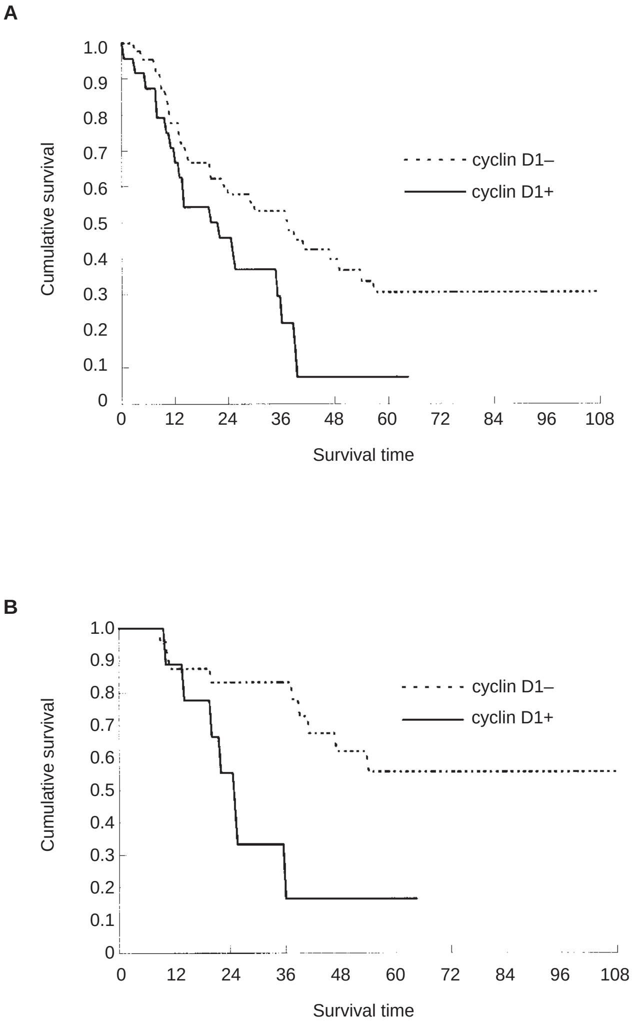

**Figure 2** Kaplan–Meier's survival curves according to immunureactivity for cyclin D1 with stages I–IIIa (**A**) and stages I–II (**B**) (log-rank; $P = 0.0299$ for stage I–IIIa, $P = 0.0066$ for stage I–II)

positive immunoreactivity respectively. There was no significant difference ($P = 0.637$).

Cyclin D1 immunoreactivity was well correlated with lymph node metastasis and the stage of the tumour. Twenty-one of 42 NSCLCs with lymph node metastasis (50%) showed positive immunoreactivity for cyclin D1. However, only three of 27 nodal metastasis-free cases (11%) disclosed cyclin D1-positive immunoreactivity. This difference was highly significant ($P = 0.002$). According to the stage of the tumour, stage I showed a lower cyclin D1-positive immunoreactivity than stages II and IIIa (10%, 53.8%, and 41.7% respectively). There were significant differences ($P = 0.006$ for stage I vs II, IIIa; $P = 0.048$ for the three groups).

### PCNA index

All tumours showed a variable degree of positive immunoreactivity for the PCNA protein in their nuclei. However, there was no significant difference in different groups according to sex, histopathological subtype, extent of the primary tumour (T status), nodal status, or stage of the tumour (ANOVA). The PCNA index

**Table 3** Multivariate Cox's analysis for independent predictors of survivals

| | β (S.E.) | *P*-value |
|---|---|---|
| Stage | 0.829 (0.314) | 0.0082 |
| Tumur status | 0.072 (0.344) | 0.8352 |
| Nodal status | 0.145 (0.443) | 0.7436 |
| Cyclin D1 | 0.277 (0.320) | 0.3878 |

β is the coefficient of the regression model. S.E., standard error.

was slightly higher in cyclin D1-positive groups in comparison with cyclin D1-negative groups (53.2% vs 47.3% respectively). However, no significant difference was present (ANOVA, $P = 0.117$).

### Survival of the patients

The overall survival ranged from 0.5 to 108 months. During the follow-up period a total of 48 patients (30.4%) died and 21 were censored. The survival of the total 69 patients according to various factors are presented in Table 2. The overall survival in the present study was significantly correlated with the extent of the primary tumour, nodal status and stage of the tumour (log-rank; $P = 0.0133$, $P = 0.0002$ and $P < 0.00001$ respectively). However, the histological type of the NSCLC and sex of the patients were not correlated with patient survival ($P = 0.1887$ and 0.6129 respectively). Sixteen of 45 cyclin D1-negative cases (35.6%) were censored. The mean survival of the cyclin D1-negative groups was significantly longer than that of the cyclin D1-positive groups ($50.1 \pm 6.4$ months vs $24.0 \pm 3.9$ months, $P = 0.029$). An analysis of the overall survival in stages I–II and stages I–IIIa revealed that cyclin D1-positive immunoreactivity was strongly associated with a poorer overall survival ($P = 0.0066$ and $P = 0.0299$ respectively; Figure 2). Multivariate analysis showed that the stage of the tumour was the strongest prognostic factor ($P = 0.0082$). However, cyclin D1-positive immunoreactivity was not significant ($P = 0.3878$) (Table 3).

### DISCUSSION

Abnormal expression of cell cycle-regulatory proteins commonly occur in various human cancers (Motokura et al, 1993; Bartkova et al, 1994; Lovec et al, 1994; Lukas et al, 1994; Wang et al, 1994). One of the most frequent derangements is altered expression of cyclin D1. Cyclin D1 is encoded by CCND1 (PRAD1 and BCL-1) located in the chromosome 11q13 region which also harbours EMSI, HSTF1 and INT2 genes. Cyclin D1 protein stimulates cyclin-dependent kinase (cdk)-mediated phosphorylation of the retinoblastoma susceptibility gene (Rb) product, which inactivates its growth-suppressive role to the transcription factor E2F. The cyclin–cdk complexes lead to transition of cell division from G1 to S phase, thus contributing to tumorigenesis in many organs by continuing cell cycle progression when they are overexpressed (Kastan and Tooze, 1997).

In the present study, we detected cyclin D1 overexpression in resected specimens of NSCLC patients using immunohistochemical methods. Twenty-four among 69 patients (34.8%) showed positive immunoreactivity for cyclin D1, which was in the range of previous studies reporting from 18% to 57.1% (Betticher et al, 1996; Kwa et al, 1996; Mate et al, 1996; Caputi et al, 1997).

 © 1999 Cancer Research Campaign

There have been several reports which showed the correlation of cyclin D1 overexpression or amplification of cyclin D1 gene (CCND1) with pathological prognostic factors and patient outcome in various human cancers. Cyclin D1 gene expression or amplification was associated with not only lymph node metastasis but also with shorter relapse-free survival in breast, head and neck, and urinary bladder cancers (Proctor et al, 1991; Schuuring et al, 1992; Jares et al, 1994; Muller et al, 1994; McIntosh et al, 1995; Åkervall et al, 1997; Shin et al, 1997). With respect to lung cancer, however, there were a few studies reported in the English literature (Betticher et al, 1996; Kwa et al, 1996; Mate et al, 1996; Caputi et al, 1997). There has been controversy, especially considering the significance of cyclin D1 overexpression on the prognosis of NSCLC patients. Betticher et al (1996) reported that cyclin D1 overexpression has been associated with some pathological parameters such as poorly differentiated histology, less infiltration of lymphocyte and a low incidence of local relapse, which suggested cyclin D1 overexpression as a good prognostic factor. On the other hand, Caputi et al (1997) demonstrated that overexpression of cyclin D1 was significantly related to short-term patient survival. In the present study, cyclin D1 overexpression in resectable NSCLCs was significantly associated with a shorter overall survival, which is compatible with the results of Caputi et al (1997). Moreover, our data revealed that overexpression of cyclin D1 was also correlated with lymph node metastasis and pathological staging. Therefore, it is suggested that cyclin D1 overexpression detected by immunohistochemical staining may be an indicator of poor prognosis in primary NSCLCs with resectability.

Furthermore, our results revealed that cyclin D1 overexpression was much more strongly correlated with the overall survival among the patients with stages I and II ($P = 0.0066$ for stages I and II vs $P = 0.0299$ for stages I–IIIa). Nine among 33 patients with stages I and II NSCLCs revealed positive immunoreactivity (27.3%), but 15 among 36 (41.7%) stage IIIa patients showed positivity. These findings suggest that overexpression of cyclin D1 can be involved in the tumorigenesis from earlier stages and may play a role in tumour progression to higher stages, which is similar to those of superficial urinary bladder cancers (Shin et al, 1997).

Our data revealed that there was no significant correlation of cyclin D1 overexpression with histological subtypes of NSCLC. This result may come from the fact that cyclin D1 overexpression is a rather conserved mechanism which is commonly involved in the tumorigenesis of various cell types of NSCLCs.

Mediastinal lymph node involvement in NSCLC is a crucial prognostic factor. Strong correlation between micrometastasis or minimal residual disease of mediastinal lymph nodes detected by immunohistochemical staining and the relapse-free survival or overall survival in stage I NSCLCs further supports the importance of lymph node involvement in the progression of NSCLCs (Izbicki et al, 1996). In this study, our data demonstrated a strong correlation of cyclin D1 overexpression with lymph node metastasis. This result suggests that metastatic potential in NSCLCs with resectability may become expressed through a way related to deregulation of cyclin D1. Mathematical studies suggest that cells probably need to accumulate at least four to six mutations to become tumorigenic. And each mutation should be required for an expansion of the mutant clone to at least a million cells (20 doublings) in which the next mutation can occur (Shay et al, 1993). These cell doublings can be achieved through the extended activity of cyclin D1 protein. Overexpression of cyclin D1 should account for their extended activity and clonal expansion of cells that have progression activity such as metastatic potential, thus implicating on the survival of the patients. Taken together with this theoretical background and our results for cyclin D1 protein, correlation of cyclin D1 overexpression with lymph node metastasis may strongly support a possible contribution of cyclin D1 overexpression to the progression and prognosis of NSCLCs.

Overexpression of cyclin D1 is believed to be correlated with increased proliferative activity, as seen in head and neck squamous cell carcinomas measured by flow cytometry (Callender et al, 1994). But it was difficult to validate correlation with other methods using bromodeoxyuridine labelling or Ki-67 (MIB-1) labelling indices (Zukerberg et al, 1995*b*). In the present study, we also measured the proliferative activity by the PCNA index using immunohistochemistry and obtained higher proliferative activity in cyclin D1-positive immunoreactive tumors than in cyclin D1-negative ones. However, no statistically significant difference was present between the two groups.

Cyclin D1 overexpression does not always mean a *sine qua non* amplification of CCND1. It has been suggested that mechanisms other than gene amplification may play a role in carcinogenesis of lung cancer including increased stability of the protein of clonal rearrangement of CCND1 gene (Zukerberg et al, 1995*a*). According to the cytogenetic study, chromosome 11q13 rearrangements were weakly correlated with immunohistochemical overexpression of cyclin D1, although it was a significant prognostic indicator of a poor outcome in head and neck cancers (Åkervall et al, 1997). In NSCLC it also remains to be elucidated whether some putative oncogenes or tumour suppressor genes within the amplicon 11q13 region other than CCND1 may be involved in the tumorigenesis.

In conclusion, our results of cyclin D1 overexpression in relation to various clinicopathological parameters suggest that overexpression of cyclin D1 is involved in tumorigenesis of NSCLC from early stage and could be a molecular marker for a poorer outcome and progression in NSCLCs with resectability. Therefore, cyclin D1 overexpression, especially in patients with stages I and II NSCLCs, may help us to determine its biological behaviour and to choose proper therapeutic modalities following tumour resection.

### REFERENCES

Adelaide J, Monges G, Derderian C, Seitz JF and Birnbaum D (1995) Oesophageal cancer and amplification of the human cyclin D gene CCND1/PRAD1. *Br J Cancer* **71**: 64–68

Åkervall JA, Michalides RJ, Mineta H, Balm A, Borg Å, Dictor MR, Jin Y, Loftus B, Mertens F and Wennerberg JP (1997) Amplification of cyclin D1 in squamous cell carcinoma of the head and neck and the prognostic value of chromosomal abnormalities and cyclin D1 overexpression. *Cancer* **79**: 380–389

Bartkova J, Lukas J, Strauss M and Bartek J (1994) Cell cycle-related variation and tissue-restricted expression of human cyclin D1 protein. *J Pathol* **172**: 237–245

Bartkova J, Lukas J, Strauss M and Bartek J (1995) Cyclin D1 oncoprotein aberrantly accumulates in malignancies of diverse histogenesis. *Oncogene* **10**: 775–778

Betticher DC, Heighway J, Hasleton PS, Altermatt HJ, Ryder WD, Cerny T and Thatcher N (1996) Prognostic significance of CCND1 (cyclin D1) overexpression in primary resected non-small-cell lung cancer. *Br J Cancer* **73**: 294–300

Callender T, el-Naggar AK, Lee MS, Frankenthaler R, Luna MA and Batsakis JG (1994) PRAD-1 (CCND1)/cyclin D1 oncogne amplification in primary head and neck squamous cell carcinoma. *Cancer* **74**: 152–158

Caputi M, De Luca L, Papaccio G, D'Aponte A, Cavallotti I, Scala P, Scarano F, Manna M, Gualdiero L and De Luca B (1997) Prognostic role of cyclin D1 in non small cell lung cancer: an immunohistochemical analysis. *Eur J Histochem* **41**: 133–138

© 1999 Cancer Research Campaign de Vita VT Jr, Hellman S and Rosenberg SA (1997) *Cancer: Principles and Practices of Oncology*. 5th edn, pp. 858–911 Lippincott: Philadelphia

Fontanini G, Vignati S, Bigini D, Mussi A, Lucchi M, Angeletti CA, Basolo F and Bevilacqua G (1995) Bcl-2 protein: a prognostic factor inversely correlated to p53 in non-small cell lung cancer. *Br J Cancer* **71**: 1003–1007

Gillett C, Fantl V, Smith R, Fisher C, Bartek J, Dickson C, Barnes D and Peters G (1994) Amplification and overexpression of cyclin D1 in breast cancer detected by immunohistochemical staining. *Cancer Res* **54**: 1812–1817

Izbicki JR, Passlick B, Hosch SB, Kubuschock B, Schneider C, Busch C, Knoefel WT, Thetter O and Pantel K (1996) Mode of spread in the early phase of lymphatic metastasis in non-small-cell lung cancer: significance of nodal micrometastasis. *J Thorac Cardiovasc Surg* **112**: 623–630

Jares P, Fernandez PL, Campo E, Nadal A, Bosch F, Aiza G, Nayach I, Traserra J and Cardesa A (1994) PRAD-1/cyclin D1 gene amplification correlates with messenger RNA overexpression and tumor progression in human laryngeal carcinomas. *Cancer Res* **54**: 4813–4817

Jiang W, Kahn SM, Tomita N, Zhang YJ, Lu SH and Weinstein IB (1992) Amplification and expression of the human cyclin D gene in esophageal cancer. *Cancer Res* **52**: 2980–2983

Kastan MB, Tooze J (1997) *Checkpoint Controls and Cancer*. Cancer Surveys, Vol 29, pp. 7–24. Cold Spring Harbor Laboratory Press: New York

Kayser K, Bulzebruk H, Probst G and Vogt-Moykopf I (1987) Retrospective and prospective tumor staging evaluating prognostic factors in operated bronchus carcinoma patients. *Cancer* **59**: 355–361

Kern JA, Schwartz DA, Nordberg JE, Weiner DB, Greene MI, Torney L and Robinson RA (1990) p185neu expression in human lung adenocarcinomas predicts shortened survival. *Cancer Res* **50**: 5184–5187

Kern JA, Slebos RJ, Top B, Rodenhuis S, Lager D, Robinson RA, Weiner D and Schwartz DA (1994) C-erbB-2 expression and codon 12 K-ras mutations both predict shortened survival for patients with pulmonary adenocarcinomas. *J Clin Invest* **93**: 516–520

Kwa HB, Michalides RJ, Dijkman JH and Mooi WJ (1996) The prognostic value of NCAM, p53 and cyclin D1 in resected non-small cell lung cancer. *Lung Cancer* **14**: 207–217

Lovec H, Sewing A, Lucibello FC, Muller R and Moroy T (1994) Oncogenic activity of cyclin D1 revealed through cooperation with Ha-ras: link between cell cycle control and malignant transformation. *Oncogene* **9**: 323–326

Lukas J, Pagano M, Staskova Z, Draetta G and Bartek J (1994) Cyclin D1 protein oscillates and is essential for cell cycle progression in human tumor cell lines. *Oncogene* **9**: 707–718

Mate JL, Ariza A, Aracil C, Lopez D, Isamat M, Perez-Piteira J and Navas-Palacios JJ (1996) Cyclin D1 overexpression in non-small cell lung carcinoma: correlation with Ki67 labelling index and poor cytoplasmic differentiation. *J Pathol* **180**: 395–399

McIntosh GG, Anderson JJ, Milton I, Steward M, Parr AH, Thomas MD, Henry JA, Angus B, Lennard TW and Horne CH (1995) Determination of the prognostic value of cyclin D1 overexpression in breast cancer. *Oncogene* **11**: 885–891

Michalides R, van Veelen N, Hart A, Loftus B, Wientjens E and Balm A (1995) Overexpression of cyclin D1 correlates with recurrence in a group of forty-seven operable squamous cell carcinomas of the head and neck. *Cancer Res* **55**: 975–978

Mitsudomi T, Steinberg SM, Oie HK, Mulshine JL, Phelps R, Viallet J, Pass H, Minna JD and Gazdar AF (1991) *ras* gene mutations in non-small cell lung cancers are associated with shortened survival irrespective of treatment intent. *Cancer Res* **51**: 4999–5002

Mitsudomi T, Oyama T, Kusano T, Osaki T, Nakanishi R and Shirakusa T (1993) Mutation of the p53 gene as a predictor of poor prognosis in patients with non-small-cell lung cancer. *J Natl Cancer Inst* **85**: 2018–2023

Motokura T and Arnold A (1993) Cyclins and oncogenesis. *Biochem Biophys Acta* **1155**: 63–78

Mountain CF (1986) A new international staging system for lung cancer. *Chest* **89**: 225S–233S

Mountain CF, Lukeman JM, Hammer SP, Chamberlain DW, Coulson WF, Page DL, Victor TA and Weiland LH (1987) Lung cancer classification: the relationship of disease extent and cell type to survival in a clinical trials population. *J Surg Oncol* **35**: 147–156

Muller D, Millon R, Lidereau R, Engelmann A, Bronner G, Flesch H, Eber M, Methlin G and Abecassis J (1994) Frequent amplification of 11q13 DNA markers associated with lymph node involvement in head and neck squamous cell carcinomas. *Eur J Cancer B Oral Oncol* **30B(2)**: 113–120

Nishida N, Fukuda Y, Komeda T, Kita R, Sando T, Furukawa M, Amenomori M, Shibagaki I, Nakao K, Ikenaga M and Ishizaki K (1994) Amplification and overexpression of the cyclin D1 gene in aggressive human hepatocellular carcinoma. *Cancer Res* **54**: 3107–3110

Naruke T, Goya T, Tsuchiya R and Suemasu K (1988) Prognosis and survival in resected lung carcinoma based on the new international staging system. *J Thorac Cardiovasc Surg* **96**: 440–447

Pezzella F, Turley H, Kuzu I, Tungekar MF, Dunnill MS, Pierce CB, Harris A, Gatter KC and Mason DY (1993) bcl-2 protein in non-small-cell lung carcinoma. *N Engl J Med* **329**: 690–694

Proctor AJ, Coombs LM, Cairns JP and Knowles MA (1991) Amplification at chromosome 11q13 in transitional cell tumours of the bladder. *Oncogene* **6**: 789–795

Rosvold E (1996) Prognostic factors for patients with non-small cell lung cancer. In: *Current Problems in Cancer. Newer Aspects in the Diagnosis, Treatment, and Prevention of Non-small-cell Lung Cancer*. Part II, Williams SD, Goulet R and Thomas G (eds), pp. 272–278. Mosby: Missouri

Schuuring E, Verhoeven E, van Tinteren H, Peterse JL, Nunnink B, Thunnissen FB, Devilee P, Cornelisse CJ, van de Vijver MJ and Mooi WJ (1992) Amplification of genes within the chromosome 11q13 region is indicative of poor prognosis in patients with operable breast cancer. *Cancer Res* **52**: 5229–5234

Shay JW, Wright WE, Brasiskyte D and Van der Haegen BA (1993) E6 of human papillomavirus type 16 can overcome the M1 stage of immortalization in human mammary epithelial cells but not in human fibroblasts. *Oncogene* **8**: 1407–1413

Shin KY, Kong G, Kim WS, Lee TY, Woo YN and Lee JD (1997) Overexpression of cyclin D1 correlates with early recurrence in superficial bladder cancers. *Br J Cancer* **75**: 1788–1792

Simpson JF, Quan DE, O'Malley F, Odom-Maryon T and Clarke PE (1997) Amplification of CCND1 and expression of its protein product, cyclin D1, in ductal carcinoma in situ of the breast. *Am J Pathol* **151**: 161–168

Tateishi M, Ishida T, Mitsudomi T, Kaneko S and Sugimachi K (1991) Prognostic value of c-erbB-2 protein expression in human lung adenocarcinoma and squamous cell carcinoma. *Eur J Cancer* **27**: 1372–1375

Wang TC, Cardiff RD, Zukerberg L, Lees E, Arnold A and Schmidt EV (1994) Mammary hyperplasia and carcinoma in MMTV-cyclin D1 transgenic mice. *Nature* **369**: 669–671

Xu HJ, Quinlan DC and Davidson AG (1994) Altered retinoblastoma protein expression and prognosis in early-stage non-small-cell lung carcinoma. *J Natl Cancer Inst* **86**: 695–699

Zhang YJ, Jiang W, Chen CJ, Lee CS, Kahn SM, Santella RM and Weinstein IB (1993) Amplification and overexpression of cyclin D1 in human hepatocellular carcinoma. *Biochem Biophys Res Commun* **196**: 1010–1016

Zukerberg LR, Yang WI, Arnold A and Harris NL (1995*a*) Cyclin D1 (PRAD1) expression in non-Hodgkin's lymphomas. Detection by immunohistochemistry. *Am J Clin Pathol* **103**: 756–760

Zukerberg LR, Yang WI, Gadd M, Thor AD, Koerner FC, Schmidt EV and Arnold A (1995*b*) Cyclin D1 (PRAD1) protein expression in breast cancer: approximately one-third of infiltrating mammary carcinomas show overexpression of the cyclin D1 oncogene. *Mod Pathol* **8**: 560–567

 *© 1999 Cancer Research Campaign*
